# Uncovering Proteins Commonly Expressed Between Heart Failure and Dementia Using Bioinformatic Tools

**DOI:** 10.3390/cimb47060437

**Published:** 2025-06-09

**Authors:** Filipa J. Costa, Rui Vitorino, Fernando Ribeiro, Ramiro D. Almeida

**Affiliations:** 1 Institute of Biomedicine (iBiMED), Department of Medical Science, University of Aveiro, 3810-193 Aveiro, Portugal; 2 Cardiovascular Research and Development Centre (UnIC), Department of Surgery and Physiology, Faculty of Medicine, University of Porto, 4200-319 Porto, Portugal; 3 Associated Laboratory for Green Chemistry (LAQV/REQUIMTE), Department of Chemistry, University of Aveiro, 3810-193 Aveiro, Portugal; 4 Institute of Biomedicine (iBiMED), School of Health Sciences, University of Aveiro, 3810-193 Aveiro, Portugal; fernando.ribeiro@ua.pt; 5 Center for Neuroscience and Cell Biology (CNC), University of Coimbra, 3004-504 Coimbra, Portugal; 6 CiBB—Centre for Innovative Biomedicine and Biotechnology, University of Coimbra, 3004-504 Coimbra, Portugal

**Keywords:** bioinformatics, VOSviewer, DisGeNET, STRING, g:Profiler, heart–brain axis

## Abstract

(1) Background: Heart failure (HF) and dementia are commonly associated with the elderly. A significant percentage of patients with HF are at high risk of cognitive decline and progression to dementia. Cognitive impairment is associated with both diseases. However, the molecules and mechanisms that affect the HF–dementia axis are poorly understood. (2) Objective: In this work, we aim to identify potential proteins that modulate HF and dementia. (3) Methods: We applied a pipeline using bioinformatic tools that robustly perform a literature search. (4) Results: Our results show that apolipoprotein E (APOE), c-reactive protein (CRP), interleukin 6 (IL6), renin (REN), and angiotensin-converting enzyme (ACE) proteins are important for maintaining homeostasis in the heart–brain axis. Additionally, deregulated levels of these proteins are associated with neuronal and cardiovascular diseases. (5) Conclusions: Our work highlights proteins that may help in understanding the pathophysiological relationship between HF and dementia. Moreover, these proteins may also be potential biomarkers and/or therapeutic targets.

## 1. Introduction

The central nervous system and cardiovascular system communicate through the sympathetic and parasympathetic nervous systems. Bidirectional interaction between the heart and brain is mediated by intrinsic cardiac neurons that interact with the brain’s vagus nerve. Substantial evidence has emerged suggesting that cardiovascular diseases may contribute to brain diseases, such as dementia and cognitive impairment [1,2].

Heart failure (HF) is a complex clinical syndrome characterized by symptoms and signs that result from structural or functional impairment of ventricular filling or ejection. These alterations lead to elevated intracardiac pressures and/or the inability of the heart to supply sufficient blood and oxygen to meet the metabolic demands of the body [3,4]. HF is one of the principal causes of hospitalization and mortality worldwide [5,6,7]. The classification of HF according to left ventricular (LV) ejection fraction (EF) is clinically important for prognosis and response to treatments. This classification is different among patients with HF with reduced EF (HFrEF) (%LVEF ≤ 40), HF with mildly reduced EF (HFmrEF) (41 ≤ %LVEF < 50), and HF with preserved EF (HFpEF) (%LVEF ≥ 50) [3,4]. Approximately half of the patients diagnosed with HF have HFrEF [8]. Moreover, HF is often seen as a consequence of other underlying cardiovascular diseases, such as coronary artery disease, or representing the final stage of several cardiac disorders [9].

As with HF, dementia is more prevalent at older ages [10,11]. The progressive decline of cognitive capacity and consequent loss of autonomy are pathological conditions that define dementia [12]. The principal causes of dementia are Alzheimer’s disease (AD) and vascular dementia [13].

Dementia has been associated with HF, since both diseases have common risk factors such as ageing, hypertension, and diabetes [14,15]; emerging evidence suggests potential direct mechanistic interactions between the cardiovascular and central nervous systems [16,17].

Evidence supports the interaction between heart and brain systems; namely, the presence of Aβ aggregates in the myocardium of AD patients compared with controls [18,19], and patients with Huntington’s disease have a higher predisposition to develop HF [20]. The heart and brain communicate through complex neural, hormonal, and inflammatory pathways, and dysregulation in one system may precipitate pathology in the other [21]. In HF patients with cognitive impairment, structural changes in the brain, such as atrophy, an increase of white matter hyper-intensities, grey matter loss, and silent cerebral infarction, have been observed [22,23,24]. Recent studies have identified shared mechanisms, including systemic inflammation, oxidative stress, neurohormonal activation, and cerebral hypoperfusion, that may explain cognitive decline in HF patients beyond general physiological deterioration [24,25]. For example, the activation of the renin-angiotensin system (RAS) in HF may contribute to neuronal injury, while inflammatory mediators such as IL6 and CRP are implicated in both neurodegeneration and cardiac dysfunction [26,27]. These insights highlight the need for an integrated view of HF and dementia pathophysiology, motivating the present study [21,24]. Furthermore, a recent systematic review with meta-analysis showed a positive association between HF and the risk of all-cause dementia (OR/RR = 1.28, 95% CI 1.15 to 1.43) [28]. Recent advances in fields such as neuroimaging technology, data analysis, or multi-omics approaches are paving the way to a clearer understanding of the imbalance of heart–brain interaction in devastating diseases such as HF and dementia [29,30].

In this work, we employed a bioinformatics-based approach to uncover proteins potentially involved in both HF and dementia. Our goal was to identify molecular links that may illuminate shared pathological pathways and reveal novel therapeutic targets. We also discuss potential pathways/mechanisms involved in the pathological process that could guide future mechanistic studies.

## 2. Methods and Materials

### 2.1. Literature Search

The relevant studies were identified by searching the Web of Science database “https://www.webofscience.com (accessed on 27 April 2022)” from its inception until 27 April 2022. The search keywords included “dementia” AND “(coronary artery disease) OR (coronary heart disease) OR (coronary atherosclerosis) OR (heart failure)”. Studies encompassing adult patients with HF or dementia were eligible. Abstracts, case reports, reviews, and articles not in English were not considered. This work is based on a pipeline published by Trindade, F. et al. [31], which describes all the steps to be implemented to carry out a literature review using bioinformatics tools.

### 2.2. Bibliometric Analysis with VOSviewer

VOSviewer software was used to identify potential molecules that are associated with HF and dementia. For this, we uploaded the literature search metadata into VOSviewer software (version 1.6.17) to detect the co-occurrence of the main keywords. Then, we manually selected the desired keywords/molecules related to both diseases. We excluded general keywords such as “diagnosis”, “risk factors”, and “population”. Next, we constructed a network linking keywords according to the strength of the association. The association strength is one method of keyword normalization used by VOSviewer and is based on a paper published by van Eck, NJ. et al. [32].

### 2.3. Gene–Disease Association Using the DisGeNET Database

The DisGeNET database “www.disgenet.org (accessed on 28 April 2022)” is used to explore specific genes associated with human diseases. In this repository, a specialized curator is responsible for carrying out the deposition of gene–disease associations, and the Unified Medical Language System (UMLS), using the concept unique identifier (CUI), identifies the diseases.

To search for genes associated with HF and dementia, we used the available web version (version 7). The UMLS CUIs associated with HF and dementia are C0018801 and C0497327, respectively. In both diseases, we only took into account genes with scores ≥ 0.10.

### 2.4. Functional Enrichment Analysis

The Venn diagram (Venny 2.1.0) [33] superimposed the molecules identified through bibliometric analysis and genes associated with two diseases. After identifying the proteins involved in both diseases, we analyzed the protein–protein interactions (PPI) and the main biological processes. For this, we used bioinformatics tools, namely STRING (version 11.5) “https://string-db.org/ (accessed on 2 May 2022)” and g:Profiler (version e106_eg53_p16_65fcd97) “https://biit.cs.ut.ee/gprofiler/gost (accessed on 16 September 2022)”. The g:Profiler core analyzes molecular functions, cellular components, biological processes, and pathways that characterize the genes of interest. This platform combines information derived from several biological databases, such as Gene Ontology (GO). The major feature of g:Profiler is to organize by statistical significance (*p*-value) the most significant keywords corresponding to the input set of genes [34].

## 3. Results

### 3.1. Bibliometric Network Analysis

**Figure 1:**
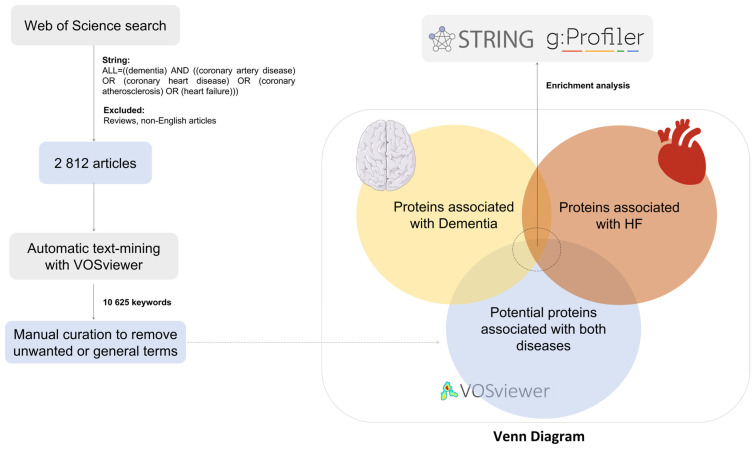
Summary scheme of methodological strategy applied. The literature search was performed using the STRING: “ALL = (dementia) AND ((coronary artery disease) OR (coronary heart disease) OR (coronary atherosclerosis) OR (heart failure))”. Reviews, editorials, case reports, and non-English articles were excluded. A total of 2812 articles were analyzed using automatic text-mining with VOSviewer. This software identifies the co-occurrence of main keywords after analyzing the entire articles downloaded. General keywords were removed by manual curation, and a network was built by association between keywords. Potential proteins associated with HF and dementia, and information available in DisGeNET about these diseases, were superimposed. After the identification of the potential proteins involved in both diseases, functional enrichment analysis was performed using the STRING and g:Profiler tools.

To investigate the relationship between HF and dementia, we performed a literature search using the Web of Science database. A total of 2812 articles were deemed suitable and downloaded into the VOSviewer software. Then, 10,625 keywords were analyzed to explore the common proteins involved in HF and dementia. The methodology is illustrated in Figure 1. Manual curation was performed to remove unwanted or generic keywords. Of these, 69 keywords were considered. Among the resulting keywords, 14 proteins were identified. The proteins are summarized in Table 1, as well as their specific entry number in the UniProt Knowledgebase (UniProtKB) and the corresponding gene identifier. Throughout the manuscript, the proteins are identified by their respective genes.

**Figure 2:**
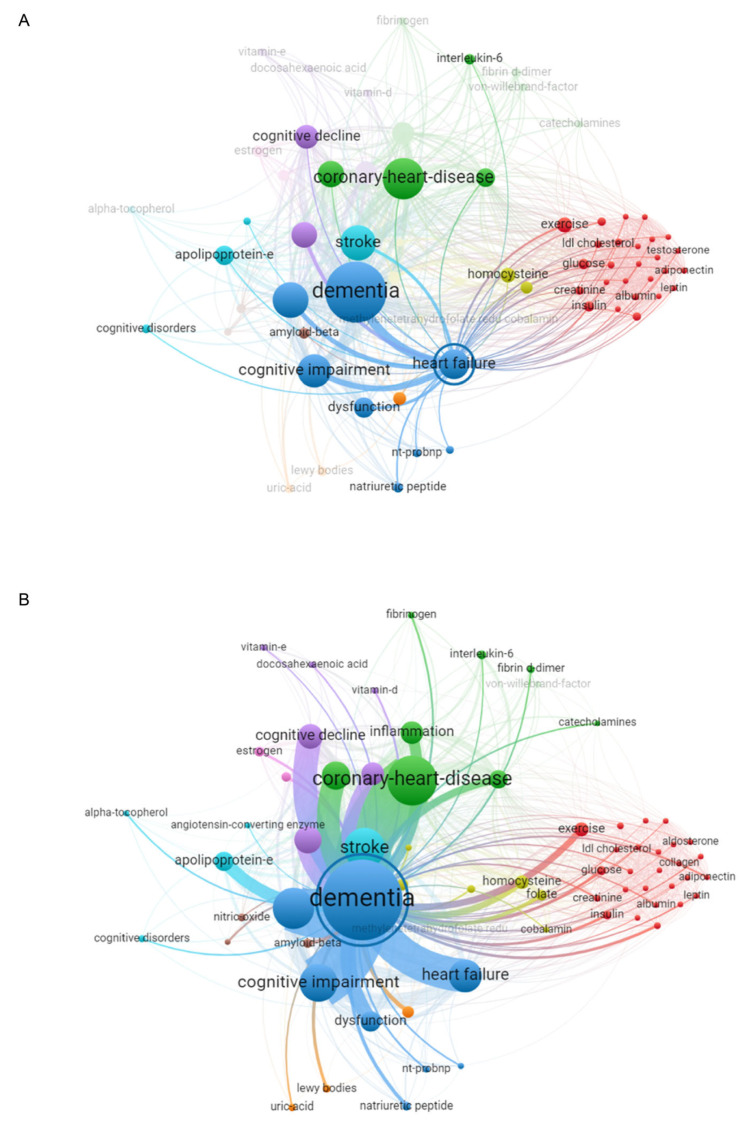
Bibliometric network highlighting the keywords associated with HF (**A**) and dementia (**B**). In the network, node size is related to frequency, and edge thickness is related to co-occurrence.

To further explore the association between HF and dementia, we constructed a network linking keywords according to the strength of the association. The node size shows the frequency of the keywords in the analyzed articles, while the edge thickness indicates the co-occurrence of the keywords (Figure 2). Examination of the highlighted keywords revealed the commonality between HF (Figure 2A) and dementia (Figure 2B), namely IL6, ACE, APOE, APP, ADIPOQ, LEP, and NPPB, suggesting that these proteins may link the two pathologies.

### 3.2. VOSviewer Results Overlaid on the DisGeNET Database

**Figure 3:**
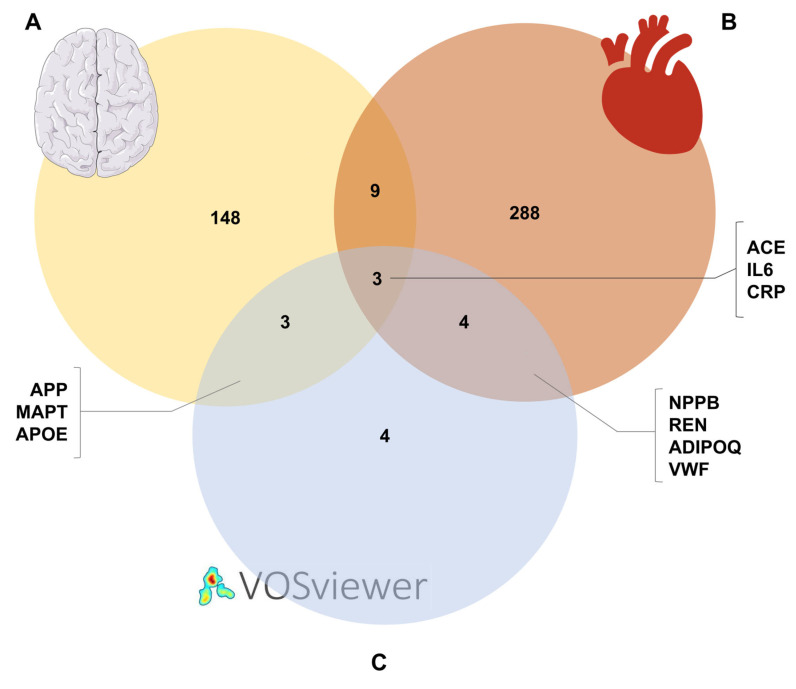
Cross-reference of results obtained through the DisGeNET database for dementia (**A**) and HF (**B**), and VOSviewer (**C**). The results of the bibliometric analysis were compared with the gene–disease association annotated in the DisGeNET repository. The UMLS CUIs for HF and dementia are C0018801 and C0497327, respectively. The Venn diagram shows the overlap of information from the two analysis methods.

We next used the DisGeNET database to support the results obtained with VOSviewer. This platform is used to explore specific genes associated with human diseases. The UMLS CUIs for HF and dementia are C0018801 and C0497327, respectively, and only genes with a cutoff score ≥ 0.10 were included in the Venn diagram (Figure 3) for both diseases. By searching the DisGeNET repository and applying the cutoff threshold, we found 163 genes associated with dementia (Figure 3A) and 304 genes related to HF (Figure 3B). Of the genes/proteins associated with dementia, six are consistent with the results obtained from VOSviewer: APP, MAPT, APOE, ACE, IL6, and CRP (Figure 3A,C). For HF, we obtained seven proteins: NPPB, REN, ADIPOQ, VWF, ACE, IL6, and CRP (Figure 3B,C). In addition, the Venn diagram shows three proteins (ACE, IL6, CRP) that are present in both diseases (Figure 3). These results highlight proteins that play a key role in the physiopathology of both HF and dementia. This finding is of particular importance, as it could explain the link between the two diseases at the molecular level.

### 3.3. Analysis of PPI and Biological Processes

**Figure 4:**
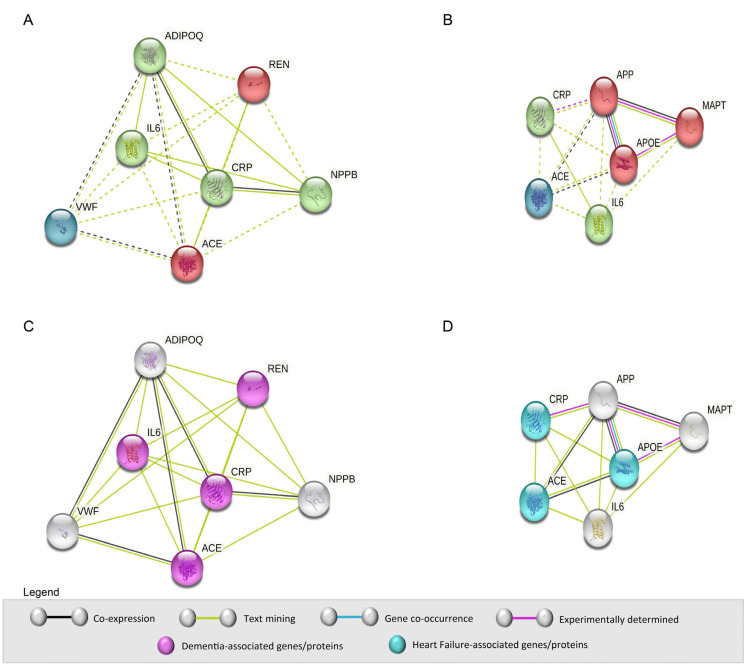
Analysis of PPI using STRING. Considering the results of the Venn diagram, seven and six proteins were identified for HF and dementia, respectively. Three of them are common to both diseases. The PPI network for each set of proteins was performed on the STRING platform. (**A**,**B**) Interaction networks built for HF (**A**) and dementia (**B**) were clustered. (**C**,**D**) Proteins associated with HF are simultaneously associated with dementia, and vice versa. Most protein interactions resulted from text-mining (light green lines).

To analyze the PPIs corresponding to the group of proteins identified for HF and dementia, we used the STRING platform. STRING integrates known information and predicts functional connections between proteins from different biological sources. Since most of the identified proteins are not present in both diseases, we performed two independent PPI analyses: the first using the proteins associated with HF (NPPB, REN, ADIPOQ, VWF, ACE, IL6, and CRP) and the second using the proteins involved in dementia (CRP, APP, MAPT, APOE, ACE, and IL6) (Figure 4).

The protein network associated with HF was determined by computational predictions and shows protein interactions from co-expression (black lines) and automated text-mining (light green lines). Based on the network, the proteins can be grouped into three clusters: ADIPOQ, IL6, CRP, and NPPB (green spheres); REN and ACE (red spheres); and VWF (blue sphere). In addition, IL6 and CRP are the hubs that interact with most proteins (Figure 4A).

Similarly, we analyzed PPI using the group of proteins associated with dementia. This group of proteins can be divided into three clusters: CRP and IL6 (green spheres); APP, MAPT, and APOE (red spheres); and ACE (blue sphere). The functional interaction between these proteins is based on biological data from literature text-mining (light green line), co-expression (black line), co-occurrence (light blue line), and experimental determination (purple line). APOE stands out at the center of this interaction network, as it interacts with all other proteins (Figure 4B).

Next, we aimed to verify which HF-related proteins are involved in dementia and explored the disease–gene association. We found that IL6, REN, CRP, and ACE play a role in dementia (purple spheres, Figure 4C). Afterwards, we verify which dementia-related proteins are involved in HF. Hence, we found that CRP, ACE, and APOE are associated with HF (light blue spheres, Figure 4D). These results demonstrate that HF and dementia have common molecular mechanisms that may contribute to the pathophysiology of both diseases.

**Figure 5:**
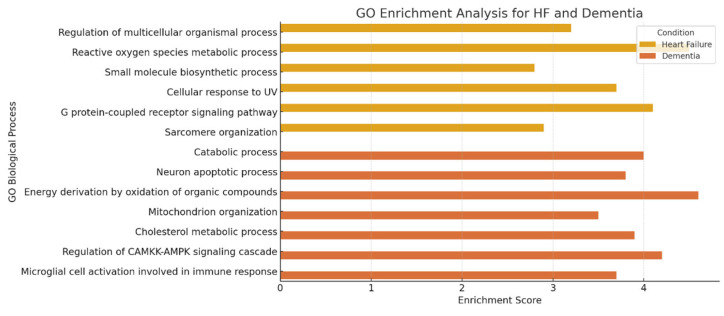
Go enrichment analysis for the protein identified with HF and dementia. Bar charts illustrate the enrichment values and the magnitude of each enriched biological process. For this analysis, we considered 304 and 163 proteins associated with HF and dementia. The bar chart provides a comparative representation of the major pathways in HF and dementia. The settings were set as default by the web server.

To determine statistical robustness and provide a more biologically meaningful interpretation of the enriched pathways for both diseases, we used the g:Profiler web server (Figure 5 and Supplementary Tables S1 and S2). For this functional analysis, 304 and 163 proteins were considered for HF and dementia, respectively. Our analysis identified several significantly enriched biological processes associated with each disease. For HF, key enriched GO terms included the regulation of multicellular organismal processes (GO:0051239), the metabolic process of reactive oxygen species (GO:0072593), and the G protein-coupled receptor signaling pathway (GO:0007187). These pathways indicate the regulation of oxidative stress, signal transduction, and structural organization of cardiac tissue, which are critical to the pathophysiology of HF. In contrast, in dementia, we observed a significant enrichment of signaling pathways associated with neurodegeneration, such as the apoptotic process of neurons (GO:0051402), the activation of microglial cells involved in the immune response (GO:0002282), and the cholesterol metabolic process (GO:0008203). These findings support the role of neuronal apoptosis, neuroinflammation, and lipid dysregulation in the progression of dementia.

Overlaps between HF and dementia, including mitochondria organization (GO:0007005) and energy generation through oxidation of organic compounds (GO:0015980), suggest common mechanisms related to metabolism and oxidative stress between these two diseases.

Table 2 summarizes the information sourced from the different bioinformatics platforms used to explore the common proteins involved in the pathophysiology of HF and dementia. In summary, the main keywords from the literature search were analyzed using VOSviewer software. The results were compared with the DisGeNET database for HF and dementia, and the Venn diagram was constructed. After identifying the proteins that simultaneously affect the pathophysiology of HF and dementia, the STRING database was used to analyze the PPI. We also used the STRING platform to explore the disease–gene association. We found an overlap of proteins between the two diseases studied. The information in Table 2 suggests that ACE, APOE, CRP, IL6, and REN proteins are the most promising candidates to explain the heart–brain interaction in a pathological context.

### 3.4. Expression Patterns Based on Literature

To further clarify the regulation of these proteins, we examined their expression patterns reported in the literature. IL6 and CRP are upregulated in both diseases due to their roles as pro-inflammatory markers contributing to systemic inflammation and disease progression [35]. APOE is a risk factor for dementia, and its effects depend on disease stage and genetic variants, particularly in Alzheimer’s disease [36]. ACE and REN, key components of the renin-angiotensin system (RAS), are upregulated in HF and contribute to vascular dysfunction [37]. However, their role in dementia is complex and varies with specific ACE gene variants, which have been associated with neurodegenerative processes [38]. These findings highlight the importance of these proteins in the pathophysiology of both diseases and may inform future research on targeted therapies.

## 4. Discussion

HF and dementia share risk factors, such as age and hypertension. Several systematic reviews and meta-analyses show that patients with HF present a high risk of cognitive decline and, consequently, dementia [28,39,40,41,42,43,44]. Cognitive decline in HF patients is mainly associated with a decrease in cerebral oxygenation as well as increased cerebral microemboli. In addition, functional changes in memory, attention, and motor capacity occur in the brain [45,46,47]. However, the identification of proteins and mechanisms that affect the HF–dementia axis is poorly understood. In this study, we used a robust text-mining and gene–disease association pipeline to uncover potential molecular links between HF and dementia. Our integrative approach consistently identified five key proteins—APOE, CRP, IL6, REN, and ACE—that may mediate shared pathological mechanisms. Notably, these proteins were found in both bibliometric and DisGeNET datasets and were further validated through STRING interaction and GO enrichment analyses.

IL6 is a pro-inflammatory factor produced by macrophages, T-cells, and adipocytes, while CRP is a product of the acute inflammatory response. The effect of these inflammatory biomarkers is associated with cognitive dysfunction, and the increase in IL6 levels is a predictor of advanced cognitive impairment [35]. IL6 can cross the blood–brain barrier and affect hippocampal regions responsible for memory and learning [48,49]. High levels of IL6 and CRP in plasma are a risk factor for dementia [50]. Furthermore, IL6 levels are upregulated in animal models with HF [51,52].

Recently, Jung et al. investigated the correlation between APOE, particularly alleles ε2 and ε4, and HF on cognitive function in adults [53], since APOE ε2 and ε4 are strongly correlated with AD [54]. The authors found that adults with HF were more likely to have cognitive impairment than participants without HF. In addition, in the sub-group of adults with HF, APOE ε4 frequency was higher in participants with mild cognitive impairment and AD when compared with individuals with normal cognition, although this difference was not statistically significant [53]. The presence of APOE ε2 was not predictive of cognitive function among participants with HF [53]. Moreover, therapeutic strategies targeting APOE have emerged as a promising approach for treating both cardiovascular and neurological diseases. Small-molecule structure correctors that can modify APOE4’s structure to resemble that of APOE3 have shown the ability to reduce mitochondrial dysfunction, decrease tau phosphorylation, and improve cellular function. Thus, by correcting APOE4’s structure, this strategy may not only improve Alzheimer’s pathology but also improve lipid metabolism, potentially lowering cardiovascular disease risk as well [55].

The primary physiological function of the RAS is the control of blood pressure and fluid homeostasis. However, RAS is also involved in neuronal and endocrine functions. This complex enzymatic pathway is present in the circulatory system and is also found in various organs, namely the brain [56]. The classical pathway of RAS consists of cleavage of the angiotensin to angiotensin I (AngI) by REN and subsequently converted to angiotensin II (AngII) by ACE [57]. AngII binds mainly to two receptors: AngII type 1 receptor and AngII type 2 receptor [58,59]. AngII receptors and ACE are pharmacologically inhibited in the treatment of patients with hypertension and HF [60,61]. In the brain, ACE variants are known to be associated with AD but have opposite functions. On the one hand, the ACE variant rs4343 has been associated with a decrease in the formation of Aβ aggregates [62,63]. On the other hand, the ACE variant rs4980 was reported to be correlated with a high risk for AD [38]. ACE inhibitors and angiotensin receptor blockers are commonly used for hypertension, although they are not currently used as front-line therapies for AD and are under investigation for their potential neuroprotective and anti-inflammatory effects [64]. Hence, the RAS pathway is one of the affected pathways in HF and dementia and may be a potential target to treat these pathologies simultaneously.

We acknowledge the limitations of in silico methods, particularly the risk of highlighting well-known, pleiotropic molecules. However, by applying strict curation criteria and multi-database validation, we aimed to enhance specificity and biological relevance. For example, IL6 and CRP are not only common inflammatory markers but are also directly implicated in neuroinflammatory cascades and cardiac remodeling [35,52]. ACE and REN, while central to cardiovascular regulation, have been linked to cognitive decline through RAS dysregulation in the brain [38]. APOE, though broadly associated with lipid metabolism and neurodegeneration, also modulates cardiovascular outcomes and emerged from our unbiased pipeline as a candidate of interest [53,55]. In conclusion, it is crucial to identify molecules and pathways that modulate the HF–dementia axis. The ACE, REN, APOE, CRP, and IL6 proteins obtained through this bioinformatic approach appear to be associated with both HF and dementia. These findings support the hypothesis that HF and dementia may share underlying biological pathways, and our approach provides a scalable framework to explore disease interactions systematically.

## Figures and Tables

**Table 1:** List of proteins resulting from bibliometric analysis.

Proteins	Gene	UniProtKB Entry
Adiponectin	*ADIPOQ*	Q15848
Amyloid β	*APP*	P05067
Angiotensin-Converting Enzyme	*ACE*	P12821
Apolipoprotein E	*APOE*	P02649
C-Reactive Protein	*CRP*	P02741
Interleukin 6	*IL6*	P05231
Leptin	*LEP*	P41159
Methylenetetrahydrofolate reductase	*MTHFR*	P42898
Microtubule-Associated Protein Tau	*MAPT*	P10636
Natriuretic Peptide B	*NPPB*	P16860
Neuron-Specific Enolase	*ENO2*	P09104
Renin	*REN*	P00797
Synaptophysin	*SYP*	P08247
Von Willebrand Factor	*VWF*	P04275

**Table 2:** Summary of proteins common to both diseases based on different bioinformatics analyses.

Proteins	VOSviewer Software	g:Profiler	Venn Diagram	STRING
ADIPOQ	✓	✓		
APP	✓	✓		
ACE	✓	✓	✓	✓
APOE	✓	✓		✓
CRP	✓	✓	✓	✓
IL6	✓	✓	✓	✓
MAPT	✓	✓		
NPPB	✓	✓		
REN	✓	✓		✓
VWF	✓	✓		

## Data Availability

All data are available in open access.

## Supplementary Materials

The following supporting information was downloaded at: https://www.mdpi.com/article/10.3390/cimb47060437/s1.

## References

[1] Zhao B., Li T., Fan Z., Yang Y., Shu J., Zhu H. (2023). Heart-brain connections: Phenotypic and genetic insights from magnetic resonance images. Science (1979).

[2] Liu W., Zhang X., Wu Z., Huang K., Yang C., Yang L. (2022). Brain–heart communication in health and diseases. Brain Res. Bull..

[3] McDonagh T.A., Metra M., Adamo M., Gardner R.S., Baumbach A., Böhm M., Burri H., Butler J., Čelutkienė J., Chioncel O. (2022). 2021 ESC Guidelines for the diagnosis and treatment of acute and chronic heart failure: Developed by the task force for the diagnosis and treatment of acute and chronic heart failure of the European Society of Cardiology (ESC). Eur. J. Heart Fail..

[4] Heidenreich P.A., Bozkurt B., Aguilar D., Allen L.A., Byun J.J., Colvin M.M., Deswal A., Drazner M.H., Dunlay S.M., Evers L.R. (2022). 2022 AHA/ACC/HFSA Guideline for the management of heart failure: A report of the American College of Cardiology/American Heart Association Joint Committee on clinical practice guidelines. Circulation.

[5] Roth G.A., Mensah G.A., Johnson C.O., Addolorato G., Ammirati E., Baddour L.M., Barengo N.C., Beaton A.Z., Benjamin E.J., Benziger C.P. (2020). Global burden of cardiovascular diseases and risk factors, 1990–2019: Update from the GBD 2019 study. J. Am. Coll. Cardiol..

[6] Metra M., Teerlink J.R. (2017). Heart failure. Lancet.

[7] Savarese G., Lund L.H. (2017). Global public health burden of heart failure. Card. Fail. Rev..

[8] Murphy S.P., Ibrahim N.E., Januzzi J.L. (2020). Heart failure with reduced ejection fraction: A review. JAMA—J. Am. Med. Assoc..

[9] Ziaeian B., Fonarow G.C. (2016). Epidemiology and aetiology of heart failure. Nat. Rev. Cardiol..

[10] Livingston G., Huntley J., Sommerlad A., Ames D., Ballard C., Banerjee S., Brayne C., Burns A., Cohen-Mansfield J., Cooper C. (2020). Dementia prevention, intervention, and care: 2020 report of the Lancet Commission. Lancet.

[11] Chen J., Dharmarajan K., Wang Y., Krumholz H.M. (2013). National trends in heart failure hospitalization stay rates, 2001 to 2009. J. Am. Coll. Cardiol..

[12] Gale S.A., Acar D., Daffner K.R. (2018). Dementia. Am. J. Med..

[13] Perl D., Pendlebury W. (1986). Neuropathology of dementia. Neurol. Clin..

[14] Vogels R.L.C., Scheltens P., Schroeder-tanka J.M., Weinstein H.C. (2007). Cognitive impairment in heart failure: A systematic review of the literature. Eur. J. Heart Fail..

[15] Festen S., De Rooij S.E. (2015). Heart failure and brain failure: Two of a kind?. Eur. J. Heart Fail..

[16] Doehner W. (2019). Dementia and the heart failure patient. Eur. Heart J. Suppl..

[17] Willis M.S., Patterson C. (2013). Proteotoxicity and cardiac dysfunction—Alzheimer’s Disease of the heart?. N. Engl. J. Med..

[18] Troncone L., Luciani M., Coggins M., Wilker E.H., Ho C.-Y., Codispoti K.E., Frosch M.P., Kayed R., del Monte F. (2016). Aβ amyloid pathology affects the hearts of patients with Alzheimer’s Disease. J. Am. Coll. Cardiol..

[19] Evangelisti A., Butler H., del Monte F. (2021). The heart of the Alzheimer’s: A mindful view of heart disease. Front. Physiol..

[20] Mielcarek M., Inuabasi L., Bondulich M.K., Muller T., Osborne G.F., Franklin S.A., Smith D.L., Neueder A., Rosinski J., Rattray I. (2014). Dysfunction of the CNS-heart axis in mouse models of Huntington’s Disease. PLoS Genet..

[21] Goyal P., Didomenico R.J., Pressler S.J., Ibeh C., White-Williams C., Allen L.A., Gorodeski E.Z., HFSA Scientific Statement Committee Members (2024). Cognitive impairment in heart failure: A heart failure Society of America Scientific statement. J. Card. Fail..

[22] Jefferson A.L., Tate D.F., Poppas A., Brickman A.M., Paul R.H., Gunstad J., Cohen R.A. (2007). Lower cardiac output is associated with greater white matter hyperintensities in older adults with cardiovascular disease. J. Am. Geriatr. Soc..

[23] Alosco M.L., Brickman A.M., Spitznagel M.B., Garcia S.L., Narkhede A., Griffith E.Y., Raz N., Cohen R., Sweet L.H., Colbert L.H. (2013). Cerebral perfusion is associated with white matter hyperintensities in older adults with heart failure. Congest. Heart Fail..

[24] Dridi H., Liu Y., Reiken S., Liu X., Argyrousi E.K., Yuan Q., Miotto M.C., Sittenfeld L., Meddar A., Soni R.K. (2023). Heart failure-induced cognitive dysfunction is mediated by intracellular Ca^2+^ leak through ryanodine receptor type 2. Nat Neurosci.

[25] Hartupee J., Mann D.L. (2017). Neurohormonal activation in heart failure with reduced ejection fraction. Nat. Rev. Cardiol..

[26] Díaz H.S., Toledo C., Andrade D.C., Marcus N.J., Del Rio R. (2020). Neuroinflammation in heart failure: New insights for an old disease. J. Physiol..

[27] Tran S., Kuruppu S., Rajapakse N.W. (2022). Chronic renin-angiotensin system activation induced neuroinflammation: Common mechanisms underlying hypertension and dementia?. J. Alzheimer’s Dis..

[28] Li J., Wu Y., Zhang D., Nie J. (2020). Associations between heart failure and risk of dementia: A PRISMA-compliant meta-analysis. Medicine.

[29] Cermakova P., Eriksdotter M., Lund L.H., Winblad B., Religa P., Religa D. (2015). Heart failure and Alzheimer′s disease. J. Intern. Med..

[30] Roher A.E. (2015). Cardiovascular system participation in Alzheimer’s Disease pathogenesis. J. Intern. Med..

[31] Trindade F., Perpétuo L., Ferreira R., Leite-Moreira A., Falcão-Pires I., Guedes S., Vitorino R. (2021). Automatic text-mining as an unbiased approach to uncover molecular associations between periodontitis and coronary artery disease. Biomarkers.

[32] van Eck N.J., Waltman L. (2009). How to normalize cooccurrence data? An analysis of some well-known similarity measures. J. Am. Soc. Inf. Sci. Technol..

[33] Oliveros J. An Interactive Tool for Comparing Lists with Venn’s Diagrams. https://bioinfogp.cnb.csic.es/tools/venny/index.html.

[34] Reimand J., Kull M., Peterson H., Hansen J., Vilo J. (2007). g:Profiler—A web-based toolset for functional profiling of gene lists from large-scale experiments. Nucleic Acids Res..

[35] Singh-Manoux A., Dugravot A., Brunner E., Kumari M., Shipley M., Elbaz A., Kivimaki M. (2014). Interleukin-6 and c-reactive protein as predictors of cognitive decline in late midlife. Neurology.

[36] Yamazaki Y., Zhao N., Caulfield T.R., Liu C.-C., Bu G. (2019). Apolipoprotein E and Alzheimer disease: Pathobiology and targeting strategies. Nat. Rev. Neurol..

[37] Jiang F., Yang J., Zhang Y., Dong M., Wang S., Zhang Q., Liu F.F., Zhang K., Zhang C. (2014). Angiotensin-converting enzyme 2 and angiotensin 1–7: Novel therapeutic targets. Nat. Rev. Cardiol..

[38] Cuddy L.K., Prokopenko D., Cunningham E.P., Brimberry R., Song P., Kirchner R., Chapman B.A., Hofmann O., Hide W., Procissi D. (2020). Aβ-accelerated neurodegeneration caused by Alzheimer’s-associated ACE variant R1279Q is rescued by angiotensin system inhibition in mice. Sci. Transl. Med..

[39] Pressler S., Kim J., Riley P., Ronis D.L., Gradus-Pizlo I. (2010). Memory dysfunction, psychomotor slowing, and decreased executive function predict mortality in patients with heart failure and low ejection fraction. J. Card. Fail..

[40] Yap N.L.X., Kor Q., Teo Y.N., Teo Y.H., Syn N.L., Evangelista L.K.M., Tan B.Y.Q., Lin W., Yeo L.L.L., Kong W.K.F. (2022). Prevalence and incidence of cognitive impairment and dementia in heart failure—A systematic review, meta-analysis and meta-regression. Hell. J. Cardiol..

[41] Vishwanath S., Qaderi V., Steves C.J., Reid C.M., Hopper I., Ryan J. (2022). Cognitive decline and risk of dementia in individuals with heart failure: A systematic review and meta-analysis. J. Card. Fail..

[42] Witt L.S., Rotter J., Stearns S.C., Gottesman R.F., Kucharska-Newton A.M., Sharrett A.R., Wruck L.M., Bressler J., Sueta C.A., Chang P.P. (2018). Heart failure and cognitive impairment in the atherosclerosis risk in communities (ARIC) study. J. Gen. Intern. Med..

[43] Wolters F.J., Segufa R.A., Darweesh S.K.L., Bos D. (2018). Coronary heart disease, heart failure, and the risk of dementia: A systematic review and meta-analysis. Alzheimer’s Dement..

[44] Jung M., Apostolova L.G., Gao S., Burney H.N., Lai D., Saykin A.J., Pressler S.J. (2024). Association of heart failure with cognitive decline and development of mild cognitive impairment and dementia. J. Cardiovasc. Nurs..

[45] Woo M.A., Kumar R., Macey P.M., Fonarow G.C., Harper R.M. (2009). Brain injury in autonomic, emotional, and cognitive regulatory area in patients with heart failure. J. Card. Fail..

[46] Woo M.A., Ogren J.A., Abouzeid C.M., Macey P.M., Sairafian K.G., Saharan P.S., Thompson P.M., Fonarow G.C., Hamilton M.A., Harper R.M. (2015). Regional hippocampal damage in heart failure. Eur. J. Heart Fail..

[47] Roy B., Woo M.A., Wang D.J.J., Fonarow G.C., Harper R.M., Kumar R. (2017). Reduced regional cerebral blood flow in patients with heart failure. Eur. J. Heart Fail..

[48] Keegan A.P., Paris D., Luis C.A., Abdullah L., Ait-Ghezala G., Beaulieu-Abdelahad D., Pryor M., Chaykin J., Crynen G., Crawford F. (2018). Plasma cytokine IL-6 levels and subjective cognitive decline: Preliminary findings. Int. J. Geriatr. Psychiatry.

[49] Monje M.L., Toda H., Palmer T.D. (2003). Inflammatory blockade restores adult hippocampal neurogenesis. Science (1979).

[50] Engelhart M.J., Geerlings M.I., Meijer J., Kiliaan A., Ruitenberg A., van Swieten J.C., Stijnen T., Hofman A., Witteman J.C.M., Breteler M.M.B. (2004). Inflammatory proteins in plasma and the risk of dementia: The Rotterdam study. Arch. Neurol..

[51] Baumgarten G., Knuefermann P., Kalra D., Gao F., Taffet G.E., Michael L., Blackshear P.J., Carballo E., Sivasubramanian N., Mann D.L. (2002). Load-dependent and -independent regulation of proinflammatory cytokine and cytokine receptor gene expression in the adult mammalian heart. Circulation.

[52] Hanna A., Frangogiannis N.G. (2020). Inflammatory cytokines and chemokines as therapeutic targets in heart failure. Cardiovasc. Drugs Ther..

[53] Jung M., Apostolova L.G., Gao S., Burney H.N., Lai D., Foroud T., Saykin A.J., Pressler S.J. (2021). Testing influences of APOE and BDNF genes and heart failure on cognitive function. Heart Lung.

[54] Chen Y., Strickland M.R., Soranno A., Holtzman D.M. (2021). Apolipoprotein E: Structural insights and links to Alzheimer disease pathogenesis. Neuron.

[55] Mahley R.W. (2016). Apolipoprotein E: From cardiovascular disease to neurodegenerative disorders. J. Mol. Med..

[56] Paul M., Mehr A.P., Kreutz R. (2006). Physiology of local renin-angiotensin systems. Physiol. Rev..

[57] Huber G., Schuster F., Raasch W. (2017). Brain renin-angiotensin system in the pathophysiology of cardiovascular diseases. Pharmacol. Res..

[58] Le D., Brown L., Malik K., Murakami S. (2021). Two opposing functions of angiotensin-converting enzyme (ACE) that links hypertension, dementia, and aging. Int. J. Mol. Sci..

[59] Carey R.M. (2017). Update on angiotensin AT2 receptors. Curr. Opin. Nephrol. Hypertens..

[60] Gavras H., Brunner H.R., Laragh J.H., Sealey J.E., Gavras I., Vukovich R.A. (1974). An angiotensin converting-enzyme inhibitor to identify and treat vasoconstrictor and volume factors in hypertensive patients. N. Engl. J. Med..

[61] Lang C.C., Struthers A.D. (2013). Targeting the renin-angiotensin-aldosterone system in heart failure. Nat. Rev. Cardiol..

[62] Zou K., Yamaguchi H., Akatsu H., Sakamoto T., Ko M., Mizoguchi K., Gong J.-S., Yu W., Yamamoto T., Kosaka K. (2007). Angiotensin-converting enzyme converts amyloid β-protein 1-42 (Aβ_1-42_) to Aβ_1-40_, and its inhibition enhances brain Aβ deposition. J. Neurosci..

[63] John S.K.K., Bailey M.H., Ridge P.G., Perry R., Wadsworth M.E., Hoyt K.L., Staley L.A., Karch C.M., Harari O., Cruchaga C. (2014). Genome-wide association study of CSF levels of 59 Alzheimer’s disease candidate proteins: Significant associations with proteins involved in amyloid processing and inflammation. PLoS Genet..

[64] Santiago T.C., Parra L., Nani J.V., Fidalgo T.M., Bradshaw N.J., Hayashi M.A.F. (2023). Angiotensin-converting enzymes as druggable features of psychiatric and neurodegenerative disorders. J. Neurochem..

