# Supplementary material for: Uncovering Proteins Commonly Expressed Between Heart Failure and Dementia Using Bioinformatic Tools
Source: Curr Issues Mol Biol. 2025 Jun 9;47(6):437. doi: 10.3390/cimb47060437 (PMC12191649; doi:10.3390/cimb47060437)
Supplement: Supplementary file 1 [file cimb-47-00437-s001.zip › cimb-3498511-Supplementary tables-main.pdf]

Supplementary tables

Supplementary Table S1. Biological process and adjusted p-values associated with HF.

Term Name	Term ID	- $log_{10}(padj)$
Regulation of multicellular organismal process	GO:0051239	72.668177
Reactive oxygen species metabolic process	GO:0072593	21.459078
Small molecule biosynthetic process	GO:0044283	11.631356
Cellular response to UV	GO:0034644	4.162858
G protein-coupled receptor signaling pathway	GO:0007187	3.955631
Regulation of membrane protein ectodomain proteolysis	GO:0051043	2.569352
Sarcomere organization	GO:0045214	2.422869

Supplementary Table S2. Biological process and adjusted p-values associated with dementia.

Term Name	Term ID	- $log_{10}(padj)$
Catabolic process	GO:0009056	19.3863948
Neuron apoptotic process	GO:0051402	15.9289146
Energy derivation by oxidation of organic compounds	GO:0015980	11.8314319
Mitochondrion organization	GO:0007005	9.6661779
Cholesterol metabolic process	GO:0008203	6.03473373
Regulation of CAMKK-AMPK signaling cascade	GO:1905289	3.15172222
Microglial cell activation involved in the immune response	GO:0002282	2.15650865
